# Comparison of cellular toxicity caused by ambient ultrafine particles and engineered metal oxide nanoparticles

**DOI:** 10.1186/s12989-015-0082-8

**Published:** 2015-03-19

**Authors:** Senlin Lu, Wenchao Zhang, Rui Zhang, Pinwei Liu, Qiangxiang Wang, Yu Shang, Minghong Wu, Ken Donaldson, Qingyue Wang

**Affiliations:** School of environmental and chemical engineering, Shanghai University, No.333, Nanchen Rd, Shanghai, 200444 China; Institute for Environmental pollution and health, Shanghai University, Shanghai, 200444 China; University of Edinburgh, Edinburgh, UK; School of Science and Engineering, Saitama University, Saitama, 338-8570 Japan

**Keywords:** Engineered nanoparticles, Ambient nanoparticles, Soluble components, Cytotoxicity

## Abstract

**Objective:**

The development of nanotechnology has spurred concerns about the health effects of exposure to nanoparticles (NPs) and ultrafine particles (UFPs). Toxicological data on NPs and UFPs may provide evidence to support the development of regulations to reduce the risk of particle exposure. We tried to provide fundamental data to determine differences in cytotoxicity induced by ambient UFPs and engineered metal oxide NPs (ZnO, NiO, and CeO_2_).

**Methods:**

UFPs were sampled by using of a nano micro-orifice uniform deposit impactor. Physicochemical characterization of the UFPs and nano metal oxide particles were studied by scanning electron microscopy and transmission electron microscopy. Cellular toxicity induced by the different particles was assessed by using of comprehensive approaches and compared after A549 cells were exposured to the particles.

**Results:**

All of the measured particles could damage A549 cells at concentrations ranging from 25 to 200 μg/mL. The lowest survival ratio and the highest lactate dehydrogenase level were caused by nano-ZnO particles, but the highest levels of intracellular reactive oxygen species (ROS) and percentages of apoptosis were observed in cells treated with the soluble fraction of ambient fine particles (PM_1.8_) at 200 μg/mL. Relatively high concentrations of anthropogenic metals, including Zn, Ni, Fe, and Cu, may be responsible for the higher toxicity of fine ambient particles compared with the ambient coarse particles and UFPs. The selected heavy metals (Zn, Ni, Fe, and Cu) were found to be located in the perinuclear and cytoplasmic areas of A549 cells. The distribution pattern of metals from ambient particles showed that distributions of the metals in A549 cells were not uniform and followed the pattern Cu > Zn > Fe > Ni, suggesting that Cu was absorbed by A549 cells more easily than the other metals.

**Conclusions:**

Metal nanoparticles oxides and UFPs at low concentration could damage to cells, but the manufactured metal oxide nanoparticles are not highly toxic to lung cells compared to environmental particles. The local concentration effect of heavy metals in A549 cells, as well as the induction of oxidative stress by the particles, may be responsible for the damage observed to the cells.

**Electronic supplementary material:**

The online version of this article (doi:10.1186/s12989-015-0082-8) contains supplementary material, which is available to authorized users.

## Background

Coarse particles are mostly deposited in the upper respiratory tract, whereas fine particles can be inhaled deep into the lung [1]. UFPs could directly injure the lung, inducing lung inflammation or translocation of inhaled particles from lung airspaces into the systemic circulation, eventually reaching other organs [2-5]. A number of studies have investigated the toxicity of ambient particles [6,7]. More recently, nanotoxicology has emerged as a new field for investigating the adverse biological outcomes of nanomaterials [2,8-10]. Because engineered nanoparticles (NPs) are now being produced in huge quantities, increased human and environmental exposure from various mechanisms, such as fugitive emission, accidental spills, and normal usage, is inevitable. Moreover, current environmental laws and occupational health guidelines are based on the nominal chemical composition of the material and seldom specify special standards for ultrafine or nanosized particles. Therefore, the potential occupational health and environmental effects of these nanosized particles are a public health concern [11].

Toxicologists have begun to focus on investigating the toxicological effects of exposure to NPs [10-14]. Because of the larger total surface area to volume ratio, small size, and other physicochemical properties (such as ability to absorb toxic metals and polycyclic aromatic hydrocarbons) of NPs, these particles can display toxicity profiles that are very different from those of larger materials of the same composition [10,15], indeed, NPs have been shown to be more toxic than coarse and fine particles [4,5]. A number of studies have focused on the physicochemical characterization and toxicity of ambient particles [16], and a mechanism through which ambient particle induce toxicity (i.e., the oxidative stress theory) has been established [3-5]. Importantly, some of the procedures and assays that are generally used to assess the adverse biological effects of ambient PM could be applied for the study of engineered NPs [2]. For example, the ability to generate ROS and oxidative injury may provide a paradigm to compare the toxic potential of NPs [14]. However, studies comparing the toxicity of exposure to ambient UFPs and engineered NPs are limited.

In this study, we compared the toxicity of metal oxide NPs (ZnO, NiO, and CeO_2_), which have been primarily used for industrial purposes, to that of ambient ultrafine, fine, and coarse particles sampled from the atmosphere in Shanghai. These results could provide fundamental data for the development of health risk assessments with respect to exposure to engineered NPs and airborne UFPs.

## Results

### Physicochemical characterization of size-resolved ambient particles

#### Mass concentrations of the size-resolved ambient particles

**Figure 1:**
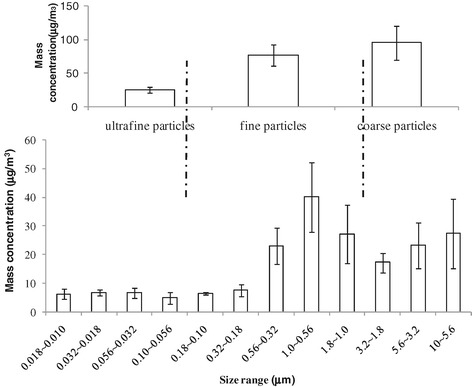
**Mass concentration of ambient size-resolved particles collected at SDK site in winter in 2009.** The sampling campaign was carried out from November 23 to December 5, 2009. The value of mass concentration of the size-resolved particles was expressed as means ± standard deviations (SDs; n = 5). The error bars indicate standard deviation.

Mass levels of the airborne particles differed according to particle size (Figure 1). The average mass concentrations of coarse particles, fine particles, and UFPs were 95.34 ± 24.92, 77.41 ± 15.6, and 25.03 ± 4.61 μg/m^3^, respectively.

### Chemical elements in the ambient particles

Next, the mass concentrations of 20 elements, i.e., Si, P, S, Cl, K, Ca, Ti, V, Cr, Mn, Fe, Ni, Cu, Zn, As, Se, Br, Rb, Sr, and Pb, in Shanghai size-segregated particles were investigated by PIXE (See Additional file 1: Table S1). The chemical elemental analysis results showed that calcium (2380.9 ng/m^3^) was the most abundant crustal element in the coarse particles, while Zn (490.48 ng/m^3^) was the most abundant trace elements in the fine particles. Si (83.66 ng/m^3^) was the most abundant element among the measured elements in the UFPs.

### Microscopic characterization of ambient particles

**Figure 2:**
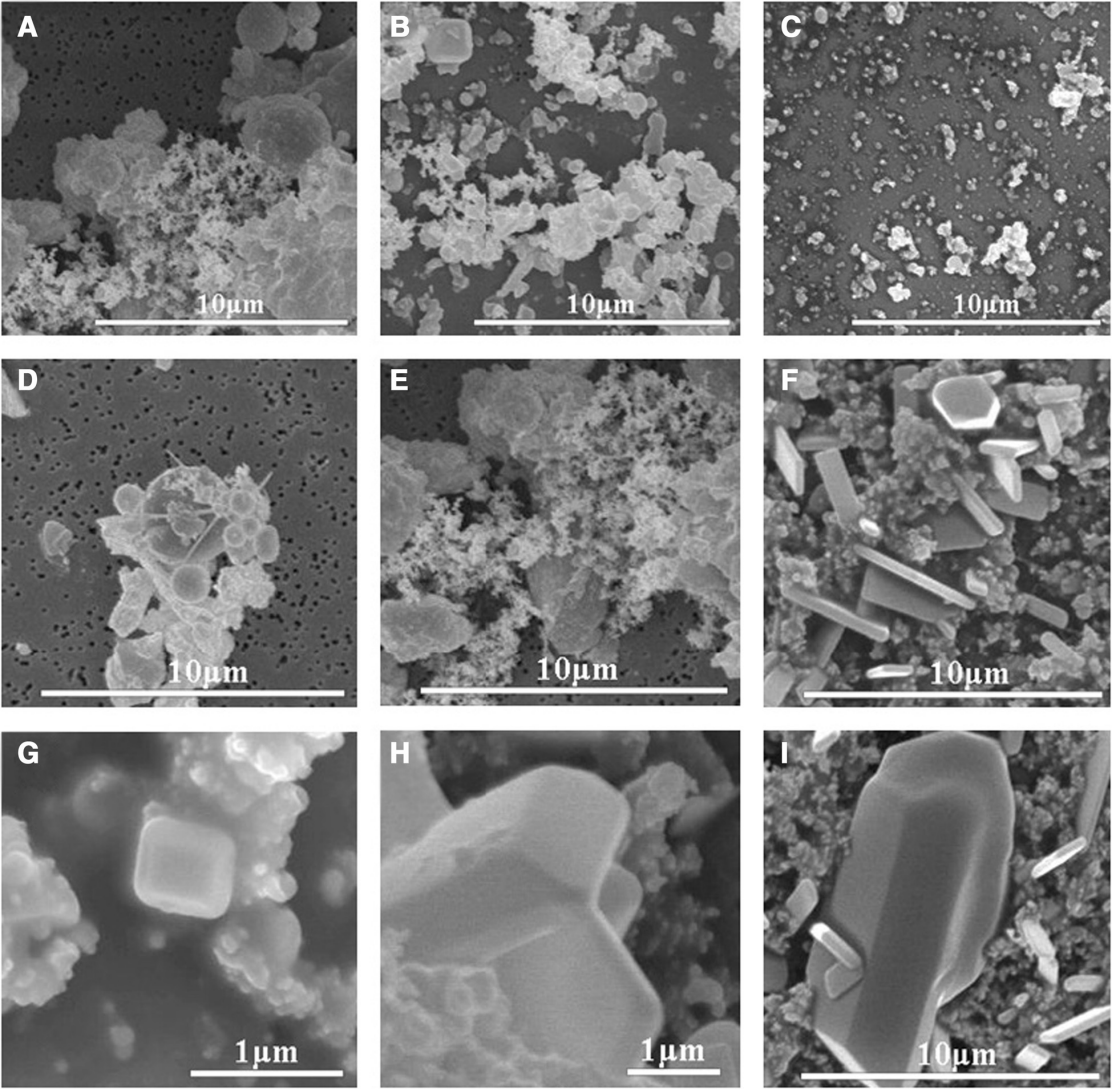
**SEM micrographs of size-segregated particles collected in Shanghai suburban atmosphere. (A)** coarse particles; **(B)** fine particles; **(C)** ultrafine particles; **(D)** fly ashes; **(E)** soot aggregates; **(F)** mineral particles containing N, O, **(G)** salts (NaCl); **(H)** and **(I)**, mineral particles containing S. Scar bar: 1 μm.

SEM revealed that particles classified into the different size categories had visibly different attributes. Based on SEM morphological characteristics (Figure 2) and EDX spectra (data not shown), individual particle types in the sampling site atmosphere could be identified as fly ash (Si, Al, and O), soot particles (carbon), regular mineral particles (S, O, Cl, and Na), and unidentified particles.

**Figure 3:**
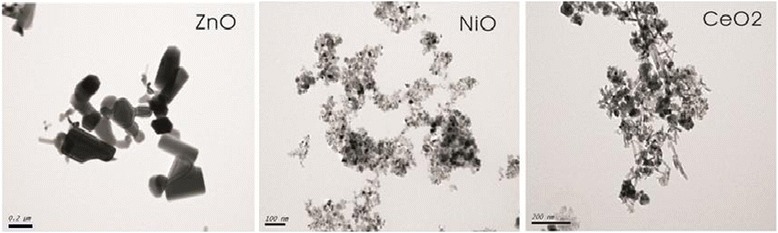
**TEM morphological characterization of metal oxide nanoparticles.** nano-ZnO had a crystal structure, while nano-NiO and -CeO_2_ were in an amorphous state.

Microcharacterization of the nano-ZnO, -NiO, and -CeO_2_ particles was performed using TEM. High-resolution TEM images revealed that nano-ZnO had a crystal structure, while nano-NiO and -CeO_2_ were in an amorphous state (Figure 3).

### MTT results

**Figure 4:**
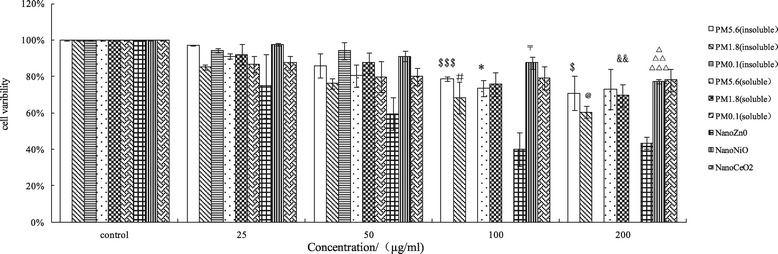
**Cell variability caused by ambient size-resolved particles and metal oxide nanoparticles ($, p < 0.05, PM**_**5.6**_ **insoluble(200 μg/ml) vs PM**_**5.6**_ **insoluble(25 μg/ml); $$$ p < 0.001, PM**_**5.6**_ **insoluble(100 μg/ml) vs PM**_**5.6**_ **insoluble(25 μg/ml); # p < 0.05, PM**_**1.8**_ **insoluble (100 μg/ml) vs PM**_**1.8**_ **insoluble (25 μg/ml); @ p < 0.05, PM**_**1.8**_ **insoluble (200 μg/ml) vs PM**_**1.8**_ insoluble (25 μg/ml) p < 0.01; *, p < 0.05, PM_**5.6**_ **soluble (100 μg/ml) vs PM**_**5.6**_ **soluble (25 μg/ml);, 〒p < 0.05, Nano NiO (100 μg/ml) vs Nano NiO,** △**Nano NiO (200 μg/ml) vs Nano NiO (100 μg/ml); **△△**p < 0.01 Nano NiO(200 μg/ml) vs Nano NiO(50 μg/ml) **△△△**Nano NiO(200 μg/ml) vs Nano NiO(25 μg/ml).**

In this experiment, due to the very low mass level of UFPs, the mass doses of the ambient UFPs were only 25 and 50 μg/mL. With these concentrations of UFPs, MTT assays were used to evaluate the proliferation of A549 cells treated with ambient size-resolved particles and metal oxide NPs. The MTT assay results showed that cell viability decreased in response to exposure to size-resolved ambient particles and NPs at 25, 50,100, and 200 μg/mL (Figure 4). For example, exposure to the insoluble and soluble fractions of coarse particles at 25 μg/mL yielded cell viabilities of 0.97 ± 0.02 and 0.91 ± 0.01, respectively, cell viability was decreased to 0.7 ± 0.09 and 0.73 ± 0.11, respectively, when the concentration of particles was increased to 200 μg/mL. Correspondingly, exposure to the insoluble and soluble fractions of fine particles decreased cell viabilities to 0.85 ± 0.01 and 0.92 ± 0.06 at 25 μg/mL and 0.6 ± 0.03 and 0.7 ± 0.06 at 200 μg/mL, respectively. Treatment with insoluble and soluble fractions of ambient UFPs at 25 and 50 μg/mL also inhibited the proliferation of A549 cells (0.94 ± 0.01 and 0.86 ± 0.04, respectively). Cell viability induced by NPs decreased in a concentration-dependent manner.

### LDH activity of the measured particles

**Figure 5:**
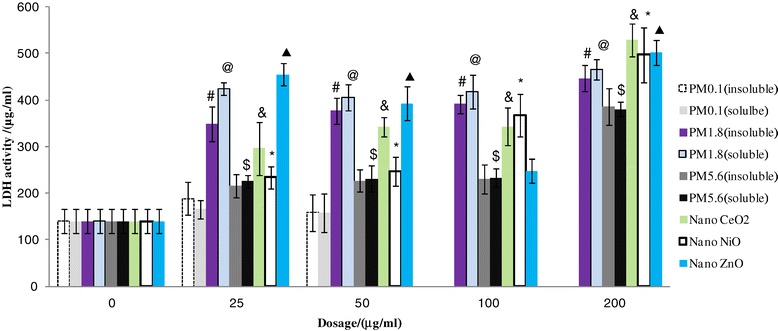
**LDH activity of A 549 cells treated with the panel of measured particles.** # p < 0.05 PM_1.8_(insoluble) vs control; @ p < 0.05 PM_1.8_(soluble) vs control; $ p < 0.05 PM_5.6_(soluble) vs control,;& p < 0.05 Nano CeO_2_ vs control; *p < 0.05 Nano NiO vs control; ▲p < 0.05 Nano ZnO vs control. Values are mean ± SD from three independent experiments.

The LDH assay results showed that the different groups of particles induced varying levels of cytotoxicity. Compared with the control group, the LDH activity in cells treated with PM_1.8_ or NPs was significantly elevated (Figure 5, *p* < 0.05). Moreover, PM_1.8_ and NPs exhibited concentration-dependent effects on LDH activity. It was noteworthy that LDH activity induced by PM_1.8_(soluble fraction) was stronger than that of PM_1.8_ (insoluble fraction).

### Intracellular ROS

**Figure 6:**
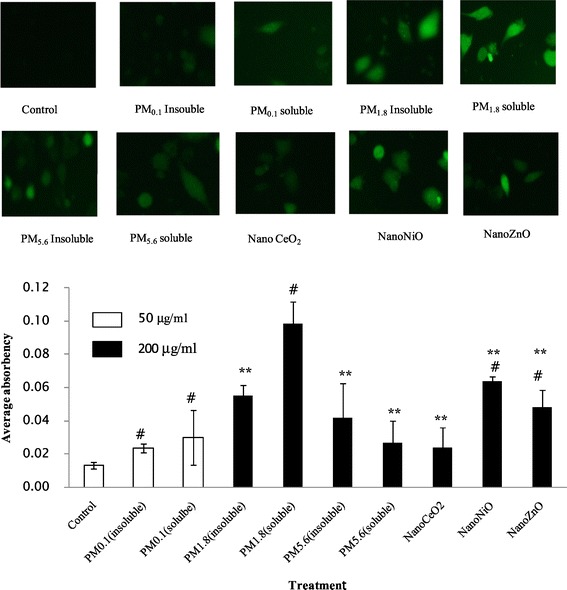
Intracellular ROS in A549 cells exposed to different components of particles, # p < 0.05 as compared to Control; **p < 0.05 as compared to PM_**1.8**_ **(soluble).** Values are mean ± SD from three independent experiments.

Similar to the results of cell viability and LDH activity, different particles exerted varying effects on intracellular ROS generation (Figure 6). Importantly, all of the particles tested in this study stimulated the induction of intracellular ROS. The highest fluorescent intensity (0.098 ± 0.013) was induced by PM_1.8_ (soluble fraction with 200 μg/mL), while the lowest fluorescent intensity (0.024 ± 0.003) was induced by ambient UFPs (insoluble fraction with 50 μg/mL).

### Induction of apoptosis in A549 cells exposure to the different particles

**Figure 7:**
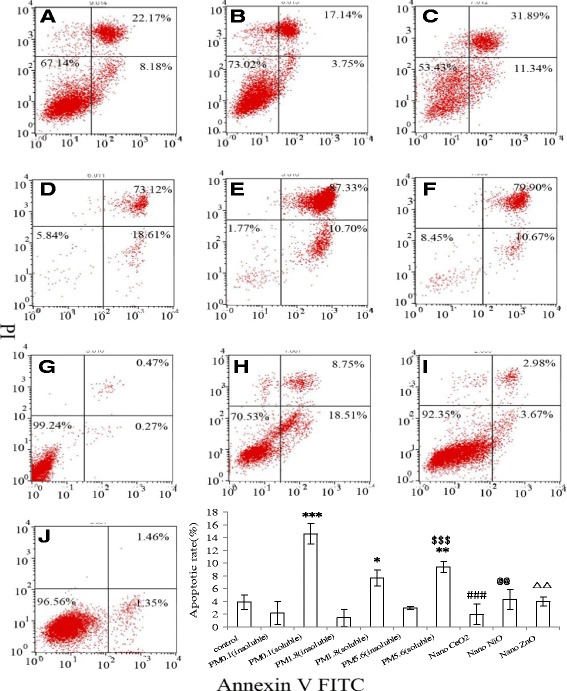
**Apoptosis of A549 cells after exposure to 200** μ**g/ml measured particles for 15 mins. A**-ultrafine particles (insoluble fraction); **B**-fine particles(insoluble fraction); **C**-coarse particles(insoluble fraction); **D**-ultrafine particles (soluble fraction); **E**-fine particles (soluble fraction); **F**-Coarse particles (soluble fraction); **G**-Nano CeO2; **H**-NiO; **I**-ZnO; **J**-control. The data was expressed as the mean ± SD of three independent experiments. *p < 0.05 PM1.8(soluble) vs control; **p < 0.01 PM5.6(soluble) vs control; ***p < 0.001 PM0.1(soluble) vs control; $$$ p < 0.001 PM0.1 (insoluble) vs PM5.6(soluble); ### p < 0.001 PM1.8 (soluble) vs Nano CeO; @@ p < 0.01 PM5.6(soluble)vs NiO; △△p < 0.01 PM5.6(soluble)vs ZnO.

Next, we analyzed the induction of apoptosis in A549 cells after a 12-h incubation with the different particle solutions. As shown in Figure 7, all the particle solutions could induce apoptosis in A549 cells. Interestingly, the soluble fractions of ambient particles induced significantly higher percentages of apoptotic cells compared the insoluble fractions and NP solutions.

### Distributions of metals in A549 cells

**Figure 8:**
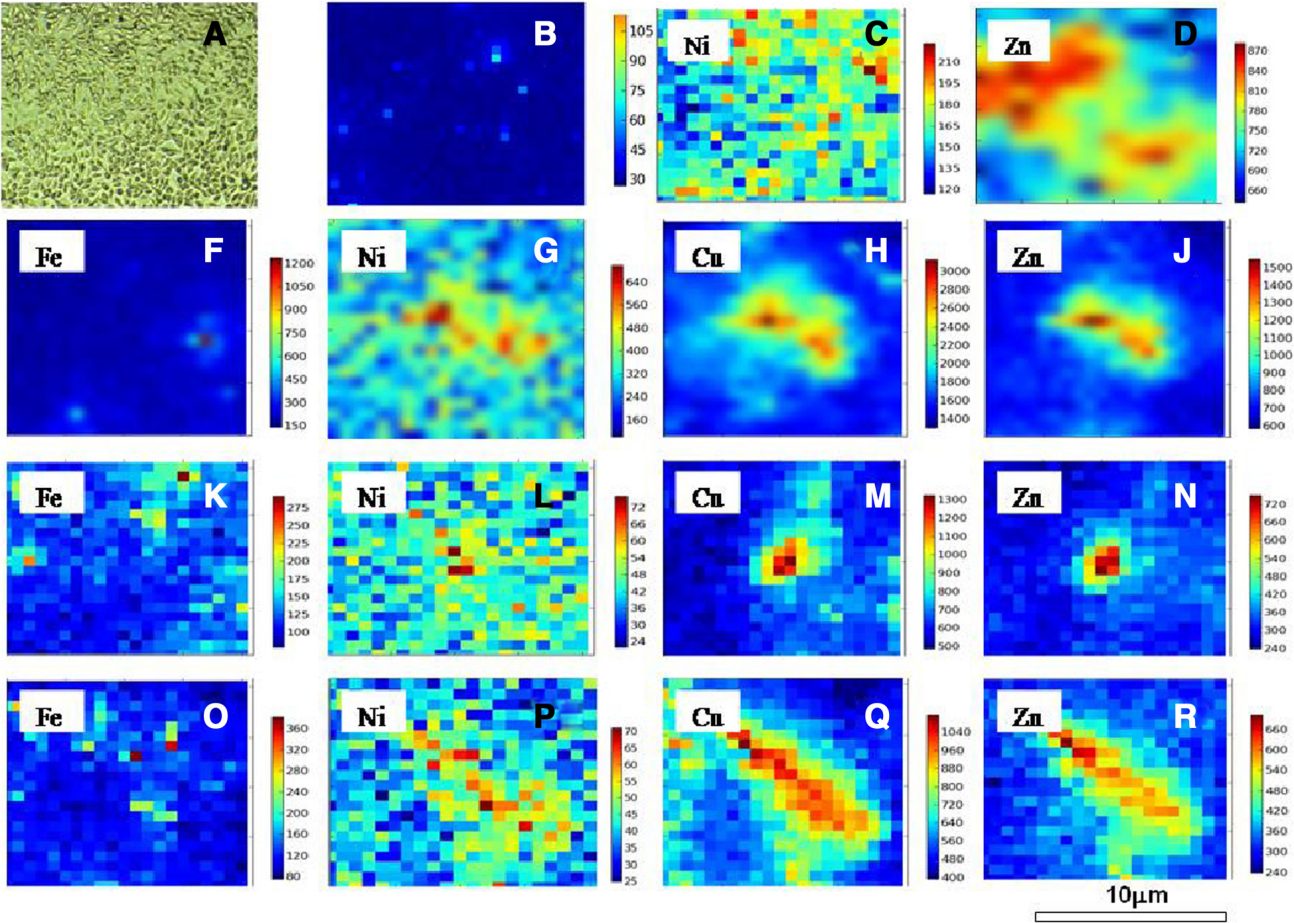
**Distribution of selected metals in subcelluar of A549. A**-cells seeded in Si_3_N_4_ plate under light microscopy (X 400); **B**-control (without any particles in cells); **C**-Nano NiO particles; **D**-Nano ZnO particles; **F** to **J**, distribution of Fe, Ni, Cu, Zn in PM _5.6_ respectively; **K** to **N**, distribution of Fe, Ni, Cu, Zn in in PM_1.8_ respectively; **O-R**, distribution of Fe, Ni, Cu, Zn in in PM_0.1_ respectively. Scar bar: 10 μm. Colour bar indicated different fluorescent intensity induced by metals in the A549 cell.

Compared with the control, Fe, Ni, Cu, and Zn exhibited differential distributions in A549 cells exposed to size-resolved ambient particles (Figure 8). The fluorescence intensity of copper (Figure 8-h) in A549 cells was the highest, followed by Zn, Ni, and Fe. For the different sizes of ambient particles (coarse, fine, ultrafine), the four heavy metals exhibited the same trends, with Cu being most abundant (followed by Zn, Fe, and Ni; Figure 8-g, h, j–r). Interestingly, among NPs, the fluorescent intensity of nano-ZnO particles was higher than that of NiO NPs in A549 cells.

## Discussion

Accumulating epidemiological and toxicological evidence has shown that there are important health risks associated with exposure to engineered NPs and ambient UFPs [9,15]. Indeed, some UFPs can be found in the heart, lungs, blood vessels, and other organs after exposure to ambient air containing nanomaterials [2,8,12,16]. While a number of recent studies and reviews have described the potential hazards and toxicities of engineered NPs, it is difficult to obtain sufficient amounts of UFPs, and few studies have investigated this type of particle. Therefore, the purpose of this study was to assess the differences in cytotoxicity induced by ambient UFPs and engineered NPs.

### Physicochemical characterization of ambient particles and NPs

Prior to toxicity studies, NPs and ambient particles should be appropriately characterized in repeated experiments in order to sufficiently characterize their toxic effects [17,18]. Therefore, we used a variety of methods, including SEM, TEM, and PIXE, to characterize the microscopic features and chemical compositions of the ambient particles and NPs. We found that Shanghai ambient particles consisted of fly ashes, soot aggregates, and mineral particles. Amorphous particles could be found in the UFP samples, while crystal particles were observed in the coarse/fine particle samples. EDX results (data not shown) demonstrated that the crystal particles consisted of Ca, S, Na, and O. Chemical analysis results (PIXE) revealed that crustal elements, i.e., Ca (2830.91 ng/m^3^), Fe (972.17 ng/m^3^), Si (961.00 ng/m^3^), and Al (414.01 ng/m^3^), were mainly distributed in the coarse particle samples, while anthropogenic elements, including Zn (490.48 ng/m^3^), Cu (36.70 ng/m^3^), and Ni (15.76 ng/m^3^), were absorbed from fine particles. Si (83.66 ng/m^3^) and Al (83.52 ng/m^3^) were the more abundant elements in the ambient UFPs, and the anthropogenic elements (such as Zn, Ni, and Cu) were found at low levels (<10 ng/m^3^) in the UFP samples.

TEM results demonstrated that nano-ZnO (90–210 nm) had a crystal structure, while nano-NiO (10–20 nm) and -CeO_2_ (20–32 nm) were in the amorphous state. These physicochemical characterizations were conducive to the following comparison of the cellular toxicity of the measured particles.

### Cytotoxicity of ambient particles and NPs

Our cell viability experiments (MTT) results demonstrated that all of the measured particles could damage A549 cells at different exposure concentrations (25, 50, 100, and 200 μg/mL). Lactate dehydrogenase (LDH) leakage assay results showed that cytoplasmic enzyme level released from the cells (after membrane damage) was in a dose-dependent manner. The lowest MTT ratio and highest LDH activity were observed for nano-ZnO particles (at the 50 μg/mL concentration), and the highest intracellular ROS intensity and apoptotic percentage were produced by the soluble fraction of ambient fine particles (PM_1.8_) at 200 μg/mL. ROS generation and oxidative stress produced by ambient particles and NPs are considered to be the main factors associated with particle toxicity [5]. Redox active metals, Fe, Cu, Ni through Fenton reaction to generate ROS and take adverse biological effects to cells, but for Zn, one of nonredox active metals, its cellular toxicity could be explained that Zn ion released from Zn containing particles or ZnO NP, was responsible for inducing inflammatory responses [19]. Deng et al. [20] also argued that the high toxicity of nano-ZnO could be explained by the mass level of Zn^2+^ in the solution. Our previous results revealed that the intensity of ROS generation by metal oxide NPs (nano-NiO and -ZnO) positively correlated with the cellular toxicity induced by the two particles; however, nano-CeO_2_ particles could also produce high levels of free radicals, its cellular toxicity was weak [14], suggesting that cellular toxicity induced by NPs was associated with both particle size and chemical composition or the types of metal ions dissolved in the particle solutions. The ROS generated by exposure to the different particles could result in various cell injuries, including membrane damage and apoptosis. Moreover, our flow cytometry analysis also demonstrated that ambient fine particles and NPs damaged the cells. Notably, the highest apoptosis rate was observed in cells treated with UFPs, rather than ambient fine particles. This phenomenon could be explained by the direct interference effect of NPs [18,21].

A number of studies have reported that NPs could translocate across the alveolar epithelial barrier and distribute into subcellular areas. SR-based techniques have been developed as a powerful technique to map the distribution of certain chemical elements in a single cell at high spatial resolution due to the extremely short wavelength of X-rays [22]. In this study, we explored the subcellular localization of selected metals with this element-sensitive, nanometer-scale resolution imaging approach to further illustrate the detailed subcellular distribution patterns of ambient particles and NPs. In the untreated cells, the metals were not distinguished from the background in most areas of the cell; however, in cells treated with NPs and ambient particles, the selected metals (Cu, Fe, Ni, and Zn) could be observed. Generally speaking, the distributions of the metals in A549 cells were not uniform, and the fluorescent intensity of the selected metals in the ambient particles followed the pattern Cu > Zn > Fe > Ni, suggesting that Cu was absorbed more easily than the other metals. We noticed that the metals were concentrated in the perinuclear and cytoplasmic areas of the cell, consistent with the conclusions reported by Chen et al. [22], who found that quantum dot NPs were concentrated in the perinuclear and marginal areas of HeLa cells. This local concentration effect of the selected metals could be responsible for the observed higher cytotoxicity of ambient fine particles than of coarse particles, i.e., the high mass concentrations of Cu, Zn, Fe, and Ni in the ambient fine particles could translocate across the alveolar epithelial barrier and distribute in subcellular areas, causing direct functional loss of these organelles. Thus, we hypothesize that this local concentration effect and the oxidative stress theory are the primary factors contributing to the effects of ambient particles and NPs on cell damage in A549 cells. Further studies are required to investigate this possibility.

## Conclusions

Our results demonstrated that all of the measured particles could damage A549 cells at different exposure concentrations (25, 50, 100, and 200 μg/mL). At the 50 μg/mL concentration, the lowest MTT ratio and highest LDH activity were observed for nano-ZnO particles. The highest intracellular ROS intensity and apoptotic percentage were produced by the soluble fraction of ambient fine particles (PM_1.8_) at 200 μg/mL. The relatively high mass concentration of anthropogenic metals, including Zn, Ni, Fe, and Cu, may be responsible for the higher toxicity of fine ambient particles compared with the ambient coarse particles and UFPs.

The selected heavy metals were concentrated in the perinuclear area and cytoplasmic area of A549 cells, as shown using SR. The distribution patterns of metals in ambient particles followed the pattern Cu > Zn > Fe > Ni, suggesting that Cu and Zn were more easily absorbed by the A549 cells. This local concentration may have important effects on cells, and oxidative stress theory may explain the damage induced in A549 cells by ambient particles and NPs. This hypothesis will need to be investigated further in future studies.

## Materials

### Sized-resolved ambient particles collected in Shanghai

The sampling site, located in Shidongkou (SDK), Shanghai (31°27′06′′; 121°24′08′′), was 2 km from Shidongkou power plant station. The sampling campaign was initiated on November 23, 2009 and was completed on December 5, 2009. A nano-MOUDI 125B sampler (MSP Co., Minneapolis, MN, USA) was installed on the fourth floor (about 10 m above ground) of the Shanghai Shengqiao Middle School building. A MOUDI sampler equipped with polycarbonate filters (47 μm in diameter) was employed to collect size-resolved ambient particles. The MOUDI 125B sampler effectively separated the PM into 13 fractions (at 50% efficiency), with the following equivalent cutoff diameters (μm): 18–10, 10–5.6, 5.6–3.2, 3.2–1.8, 1.8–1.0, 1.0–0.56, 0.56–0.32, 0.32–0.18, 0.18–0.1, 0.1–0.056, 0.056–0.032, 0.032–0.018, 0.018–0.010 μm. The flow rate of the MOUDI 125B sampler was 10 L/min. PM samples were collected onto polycarbonate filters (Millipore, UK) with pore sizes of 0.6 μm. The sampling time was 48 h. The filters were kept in a desiccator until use.

### Metal oxide NPs

Three types of metal oxide NPs, CeO_2_, NiO, and ZnO, were purchased from Nanamor Co., USA. The sizes of the engineered nano-CeO_2_, -NiO, and -ZnO particles were 20–32, 10–20, and 90–210 nm, respectively.

## Methods

### Physicochemical characterization of ambient particles and NPs

#### Microscopic characterization of ambient particles and NPs

Ambient particles were observed using a scanning electron microscope (SEM; JSM-6700 F, Japan) as described by Lu et al. [23]. Briefly, SEM images were obtained on an SEM equipped with an energy-dispersive X-ray system (EDX) and a Si (Li) detector, which allows X-ray detection from elements higher than carbonate (Z > 6). Operation conditions were as follows: 20 keV accelerating voltage and 600 pA beam current, with spectral acquisition times of 30-100 s.

Microscopic characterization of NPs was observed using a transmission electron microscope (TEM). For TEM observations, NPs were dispersed in ethanol by sonication, and a droplet of ethanol was then removed using a pipette and placed on the TEM grid. After the grid dried at room temperature, the NPs were observed under a JEM-2010 F TEM (Japan). The TEM was operated at 200 kV, and high-resolution TEM images were acquired.

### Analysis of chemical elements

Chemical elements of the size-resolved particles were analyzed using proton-induced X-ray emission analysis (PIXE), as previously described [24]. Briefly, the filtered samples were cut into strips (width × length = 6 × 3 mm^2^) and aligned in parallel on a plastic frame. About 1 mm was left blank between each sample, and a blank of about 10 mm was left in front of the first sample; in this position, we placed a fluorescent paper in order to perform appropriate instrumental adjustments. The collected samples were analyzed by PIXE (General Ionex Corp., USA) at the Institute of Low Energy Nuclear Physics, Beijing Normal University. This PIXE analyzer has been verified to be reliable by international standard intercomparisons [25,26]. For each sample, concentrations of 20 elements were determined, including Mg, Si, P, S, Cl, K, Ca, Ti, V, Cr, Mn, Fe, Ni, Cu, Zn, As, Se, Br, and Pb. One blank filter was also analyzed for corrections of multi-element concentrations.

The chemical elements of CeO_2_, NiO, and ZnO particles in water were previously analyzed [14].

### Particle solution preparation

The ambient particles were divided into three groups based on their size range: PM_10–5.6_, PM_5.6–3.2_, and PM_3.2–1.8_ were combined as coarse particles; PM_1.0–0.56_, PM_0.56–0.32_, PM_0.32–0.18_, and PM_0.18–0.1_ were combined as fine particles; and PM_0.1–0.056_, PM_0.056–0.032_, PM_0.032–0.018_, and PM_0.018–0.01_ were combined as UFPs.

Filters were immersed in 5 mL deionized water in Eppendorf tubes for 1 h and then sonicated (300 W, 30 kHz) for 30 min. The filters were then removed from the tubes and allowed to air dry at room temperature. The mass dosage of the particle solution was determined by measuring the difference in filter weights before and after immersing in water. Soluble fractions of the particles were achieved as follows. The stock solution was centrifuged at 3000 rpm for 30 min, and the supernatant (2 mL) was then carefully removed as the soluble fraction. The remaining 3 mL was dried by condensation, and after the procedure, 3 mL of deionized water was added into the tube as the particle insoluble fraction. The particle stock solution was diluted to 25, 50, 100, or 200 μg/mL for subsequent experiments.

Solutions of engineered NPs were prepared by the following procedure. First, 0.2 g NPs were dissolved into 100 mL sterile deionized water in a measuring flask and sonicated for 1 h at room temperature. The stable suspension of NPs was used immediately. The 200 μg/mL particle solution was diluted to 100, 50, and 25 μg/mL with sterile deionized water for cell exposure experiments.

### Cell culture

The type II human alveolar epithelial cell line A549 was maintained in continuous culture in Dulbecco’s modified Eagle’s medium (DMEM) supplemented with 10% heat-inactivated fetal bovine serum (FCS), 2 mM glutamate, 100 IU/mL streptomycin, and 100 μg/mL penicillin. Cells grew to confluence at 37°C in a humidified atmosphere containing 5% CO_2_ and were washed with phosphate-buffered saline (PBS), followed by harvesting with trypsin-EDTA.

### MTT assay

For analysis of cell proliferation, 1 × 10^3^ to 1 × 10^4^ cells were seeded in each well in 96-well culture plates and allowed to attach for 24 h. The cells were then washed with D-Hank’s three times and exposed to 100 μL of the ambient particle (coarse/fine/ultrafine) solution or difference concentrations of NPs (25, 50, 100, and 200 μg/mL) for 4 h. Next, 10 μL of 5 mg/mL MTT solution was added to the culture medium and incubated for 4 h at 37°C. The MTT reaction was terminated by addition of 150 μL of dimethyl sulfoxide (DMSO), and the absorbance at 490 nm was recorded. Cells were treated in triplicate, and the experiment was repeated three times.

### Lactate dehydrogenase (LDH) leakage assay

Cytotoxicity was assessed by LDH release as described by An et al. [27]. Briefly, LDH enzyme activity was assayed by colorimetry at 440 nm using a Quant microplate spectrophotometer (BioTek Instruments, Winooski, VT, USA). LDH leakage assays were performed to measure the cytotoxicity of the measured particles according to the manufacturer’s protocol. The LDH activity was determined with following formula: LDH activity (U/L) = [(sample OD − control OD)/(standard OD − blank OD)] × standard concentration × dilution factor × 1000. All experiments were carried out in triplicate and repeated three times.

### Measurement of intracellular ROS

The levels of intracellular ROS were determined by the change in fluorescence resulting from the oxidation of the fluorescent probe 2′,7′-dichlorofluorescein diacetate (DCFH-DA). Cells were seeded in 6-well plates at a density of 1 × 10^5^ cells/mL and grown to confluence in media containing 10% fetal bovine serum (FBS). After exposure to ambient UFPs (50 μg/mL, the maximum dosage of the UFPs we can prepare), ambient coarse particles, ambient fine particles, or nano particles (200 μg/mL), cells were treated with 10 μM DCFH-DA. The plates were incubated at 37°C for 30 min in the dark, and cells were then washed twice with warm D-Hank’s solution and evaluated under a fluorescence microscope (Olympus BX-51, Japan). The intensity of fluorescence was analyzed by Image-pro plus 6.0 software. Serum-free culture was used as negative control.

### Annexin V-FITC/propidium iodide (PI) apoptosis assay

The quantification of apoptosis induced by silica particles in A549 cells was measured by flow cytometry (FCM, Becton Dickinson, USA) with Annexin V-FITC/PI double staining as described by Deng et al. [20]. Briefly, the cells were harvested after 12 h of exposure to ambient UFPs (50 μg/mL), ambient coarse particles, ambient fine particles, or NPs (200 μg/mL); washed twice with cold PBS (0.15 M, pH = 7.2); and resuspended to 1 × 10^6^ cells/mL in binding buffer. Then, 100 μL of cells was transferred to a 5-mL culture tube, and 5 μL of FITC-conjugated Annexin V (Annexin V-FITC) and 5 μL PI were added at room temperature in the dark. After incubation for 15 min at room temperature in the dark, stained A549 cells were diluted by the same binding buffer and directly analyzed by fluorescence-activated cell sorting (FACS, FACSCalibur, BD Biosciences, USA) according to the manufacturer’s instructions. At least 10,000 cells were collected and detected by flow cytometry, and the percentages of apoptotic cells were analyzed by FACS Diva 4.1 software.

### Distribution of selected metals in A549 cells

For analysis of the distribution of selected metals in cells, we used the methods described by Carter et al. [28], with modifications. A drop of A549 cell solution containing 10% FCS was removed onto a sterilized Si_3_N_4_ crystal plate (attached to the bottom of a 24-well plate) using of pipette, and 5 mL DMEM was added into the wells. The plate was then incubated in a humidified atmosphere containing 5% CO_2_ at 37°C for 24 h. After the Si_3_N_4_ plate (with cells attached) was washed with D-Hank’s three times, 5 μL of the particle solution (ambient particles, NPs; 25 μg/mL) was added into the wells. Then, the Si_3_N_4_ plate was kept in the incubator for 4 h. The plate was washed with D-Hank’s, and the cells were fixed on the Si_3_N_4_ with alcohol (95%).

The distribution of metal in the cells was analyzed with SR-μXRF on a beamline BL15U instrument at Shanghai Synchrotron Radiation Facility (Shanghai, China) (Qiu et al.) [29]. The energy of the storage ring was 3.5 GeV and the beam current was 200–300 mA. Continuous synchrotron X-rays were monochromatized by an Si (111) double crystal. A monochromatic X-ray beam with a photon energy of 10 keV was used to excite the samples. The cross-section of the beam irradiating on the samples was adjusted to about 2 × 2 μm^2^. The sample was placed at a 45° angle to the incident X-ray beam, and X-ray fluorescence was detected with a 50 mm^2^ silicon drift detector (Vortex, USA) oriented at a 90° angle to the incident beam. A light microscope was coupled to a computer for sample viewing. The sample platform was moved by a motorized *x*-*y* mapping stage. The distributions of selected metals (Ni, Fe, Cu, and Zn) in the cells were continuously scanned at a step of 2 μm for both the *x* and *y* directions. Each spot was irradiated for 100 s. The data were analyzed by Plot software, and the differential metal distribution map was exported.

### Statistical analysis

The data for mass concentration of ambient particles was analyzed by Excel and expressed as means ± standard deviations (SDs; n = 5). The MTT assays, LDH assays, and intracellular ROS data were analyzed using SPSS software version 13.0 (SPSS Inc., Chicago, IL, USA), and data were expressed as means ± SDs. Statistical significance was determined by using one-way analysis of variance (ANOVA). Differences with *p* values of less than 0.05 were considered significant.

### Supporting information

More details on mass level of chemical elements in the size-resolved particles were listed in supplementary table.

## Additional file

Additional file 1: Table S1. Chemical elements in ultra/fine/coarse particles (ng/m^3^).

## Abbreviations

LDH: Lactate dehydrogenase
MTT: 3-(4,5-dimethyl-2-thiazolyl)-2,5-diphenyl-2-H-tetrazolium bromide
PIXE: Proton-induced X-ray emission analysis
PI: Propidium iodide
NPs: Nanoparticles
ROS: Reactive oxygen species
SD: Standard deviation
SEM: Scanning electron microscopy
SR-μXRF: Synchrotron radiation X-ray fluorescence micro-spectroscopy
TEM: Transmission electron microscopy
UFPs: Ultrafine particles

## References

[1] Kleinstreuer C, Zhang Z, Li Z (2008). Modeling airflow and particle transport/deposition in pulmonary airways. Respir Physiol Neurobiol.

[2] Xia T, Kovochich M, Brant J, Hotze M, Sempf J, Oberley T (2006). Comparison of the abilities of ambient and manufactured nanoparticles to induce cellular toxicity according to an oxidative stress paradigm. Nano Lett.

[3] Donaldson K, Beswick PH, Gilmour PS (1996). Free radical activity associated with the surface of particles: a unifying factor in determining biological activity?. Toxicol Lett.

[4] Donaldson K, Brown DM, Mitchell C, Dineva M, Beswick PH, Gilmour P (1997). Free radical activity of PM10: iron-mediated generation of hydroxyl radicals. Environ Health Perspect.

[5] Donaldson K, Tran L, Jimenez LA, Duffin R, Newby DE, Mills N (2005). Combustion-derived nanoparticles: a review of their toxicology following inhalation exposure. Part Fibre Toxicol.

[6] Donaldson K, Li X, MacNee W (1998). Ultrafine (Nanometer) particle mediated lung injury. J Aerosol Sci.

[7] Donaldson K, Stone V, Borm P, Jimenez L, Gilmour P, Schins R (2003). Oxidative stress and calcium signaling in the adverse effects of environmental particles (PM10). Free Radic Biol Med.

[8] Oberdorster G, Oberdorster E, Oberdorster J (2009). Nanotoxicology: an emerging discipline evolving from studies of ultrafine particles. Environ Health Perspect.

[9] Yacobi N, Fazllolahi F, Kim Y, Sipos A, Borok Z, Kim K (2011). Nanomaterial interactions with and trafficking across the lung alveolar epithelial barrier: implications for health effects of air-pollution particles. Air Qual Atmos Health.

[10] Cassee FR, Mills NL, Newby DE (2011). Cardiovascular Effects O Inhaled Ultrafine and Nano-Sized Particles.

[11] Veranth J, Kaser E, Veranth M, Koch M, Yost G. Cytokine response of human lung cells (BEAS-2B) treated with micro-sized and nanoparticles of metal oxides compared to soil dusts. Part Fibre Toxicol. 2007. doi:10.1186/1743-8977-4-2. 10.1186/1743-8977-4-2 PMC1821039 17326846

[12] Oberdörster G, Sharp Z, Atudorei V, Elder A, Gelein R, Kreyling W (2004). Translocation of inhaled ultrafine particles to the brain. Inhal Toxicol.

[13] Poland C, Duffin R, Kinloch I, Maynard A, Wallace W, Seaton A (2008). Carbon nanotubes introduced into the abdominal cavity of mice show asbestos-like pathogencity in a pilot study. Nat Nanotechnol.

[14] Lu S, Duffin R, Poland C, Daly P, Murphy F, Drost E (2009). Efficacy of short-term in vitro assay for predicting the potential of a panel of metal oxide nanoparticles to cause lung inflammation. Environ Health Perspect.

[15] Mills NL, Donaldson K, Hadoke PW, Boon NA, MacNee W, Cassee FR (2009). Adverse cardiovascular effects of air pollution. Nature.

[16] Xia T, Kovochich M, Liong M, Meng H, Kabehie S, George S (2009). Polyethyleneimine coating enhances the cellular uptake of mesoporous silica nanoparticles and allows safe delivery of siRNA and DNA constructs. ACS Nano.

[17] Balbus J, Maynard A, Colvin V, Castronova V, Daston G, Denison R (2007). Meeting report: hazard assessment for nanoparticles—report from an interdisciplinary workshop. Environ Health Perspect.

[18] Li Y, Sun L, Jin M, Du Z, Liu X, Guo C (2011). Size-dependent cytotoxicity of amorphous silica nanoparticles in human hepatoma HepG2 cells. Toxicol In Vitro.

[19] Vandebriel RJ, Jong D (2012). A review of mammalian toxicity of ZnO nanoparticles. Nanotechnol Sci Appl.

[20] Deng X, Luan Q, Chen W, Wang Y, Wu M, Zhang H, Jiao Z. Nanosized zinc oxide particles induce neural stem cell apoptosis. Nanotechnology. 2009:20. doi:10.1088/0957-4484/20/11/115101. 10.1088/0957-4484/20/11/115101 19420431

[21] Trouliller B, Reliene R, Westbrook A, Solaimani P, Schiestl R (2009). Titanium dioxide nanoparticles induce DNA damage and genetic instability in vivo in mice. Cancer Res.

[22] Chen N, He Y, Su Y, Li X, Huang Q, Wang H (2012). The cytotoxicity of cadmium-based quantum dots. Biomaterials.

[23] Lu S, Shao L, Wu M, Jiao Z (2006). Mineralogical characterization of airborne individual particulates in Beijing PM10. J Environ Sci.

[24] Lu S, Shao L, Wu M, Jiao Z, Chen X (2007). Chemical elements and their source apportionment of PM10 in Beijing urban atmosphere. Environ Monit Assess.

[25] Zhu G, Wang G (1998). Investigation of the particulate derived from indigenous zinc smelting using PIXE analytical technique. Nucl InstrumMethods Phys Res B.

[26] Zhu G, Wang G (2000). International comparison of PIXE analysis results. Climate Environ Res.

[27] An J, Yin L, Shang Y, Zhong Y, Zhang X, Wu M (2011). The combined effects of BDE47 and BaP on oxidatively generated DNA damage in L02 cells and the possible molecular mechanism. Mutat Res.

[28] Carter E, Rayner B, Mcleod A, Wu L, Marshall C, Levina A (2010). Silicon nitride as a versatile growth substrate for microspectroscopic imaging and mapping of individual cells. Molecular Biosystems.

[29] Qiu J, Deng B, Yang Q, Yan F, Li A, Yu X (2011). Internal elemental image by scanning X-ray fluorescence microtomography at the hard X-ray microprobe beamline of the SSRF: Preliminary experimental results. Nucl InstrumMethods Phys Res B.

