# Supplementary material for: Comparison of cellular toxicity caused by ambient ultrafine particles and engineered metal oxide nanoparticles
Source: Part Fibre Toxicol. 2015 Mar 19;12:5. doi: 10.1186/s12989-015-0082-8 (PMC4412114; doi:10.1186/s12989-015-0082-8)
Supplement: Additional file 1: Table S1. — Chemical elements in ultra/fine/coarse particles (ng/m3). [file 12989_2015_82_MOESM1_ESM.doc]

Supplementary table

Table 1 Chemical elements in ultra/fine/coarse particles (ng/m^3^)

|  | 0.018~0.010 | 0.032~0.018 | 0.056~0.032 | 0.10~0.056 | UPFs | 0.18~0.10 | 0.32~0.18 | 0.56~0.32 | 1.0~0.56 | 1.8~1.0 | fine particles | 3.2~1.8 | 5.6~3.2 | 10~5.6 | coarse particles |
| --- | --- | --- | --- | --- | --- | --- | --- | --- | --- | --- | --- | --- | --- | --- | --- |
| Mg | 20.91 | 0.00 | 28.18 | 0.00 | 49.09 | 0.00 | 0.00 | 38.39 | 34.07 | 43.53 | 115.98 | 0.00 | 90.77 | 0.00 | 90.77 |
| Al | 49.47 | 8.39 | 25.66 | 0.00 | 83.52 | 0.00 | 0.00 | 0.00 | 16.87 | 104.26 | 121.13 | 89.47 | 310.31 | 14.23 | 414.01 |
| Si | 54.45 | 9.74 | 17.31 | 2.16 | 83.66 | 1.96 | 8.25 | 20.36 | 74.17 | 267.65 | 372.39 | 231.43 | 685.51 | 44.05 | 961.00 |
| P | 41.35 | 0.00 | 6.05 | 0.00 | 47.39 | 0.00 | 0.00 | 0.00 | 6.32 | 11.72 | 18.04 | 5.76 | 0.00 | 0.00 | 5.76 |
| S | 31.73 | 7.60 | 11.67 | 8.15 | 59.15 | 10.54 | 46.32 | 192.68 | 534.07 | 520.33 | 1303.94 | 71.88 | 230.01 | 54.95 | 356.84 |
| Cl | 12.17 | 6.40 | 1.82 | 1.55 | 21.93 | 0.00 | 5.70 | 142.96 | 236.14 | 275.27 | 660.07 | 39.21 | 152.37 | 109.40 | 300.98 |
| K | 1.41 | 4.37 | 2.71 | 2.10 | 10.59 | 3.56 | 12.47 | 79.87 | 121.23 | 130.70 | 347.83 | 36.97 | 119.25 | 51.02 | 207.24 |
| Ca | 3.63 | 1.67 | 1.07 | 1.07 | 7.44 | 4.01 | 6.03 | 12.83 | 40.45 | 380.28 | 443.60 | 524.23 | 1384.15 | 472.53 | 2380.91 |
| Fe | 1.73 | 4.43 | 1.09 | 3.43 | 10.68 | 7.46 | 19.68 | 82.72 | 192.43 | 323.07 | 625.35 | 212.43 | 559.15 | 200.60 | 972.17 |
| Sc | 0.79 | 0.00 | 4.07 | 0.00 | 4.86 | 0.00 | 1.00 | 1.18 | 0.00 | 0.00 | 2.19 | 5.70 | 9.36 | 5.24 | 20.30 |
| Ti | 0.58 | 0.99 | 0.00 | 0.00 | 1.57 | 0.64 | 0.00 | 0.90 | 5.13 | 21.91 | 28.57 | 11.81 | 41.38 | 12.77 | 65.95 |
| V | 0.00 | 0.00 | 0.00 | 0.00 | 0.00 | 0.00 | 0.38 | 3.87 | 0.00 | 0.00 | 4.26 | 1.87 | 3.24 | 0.00 | 5.11 |
| Cr | 0.00 | 0.00 | 0.92 | 0.00 | 0.92 | 1.70 | 2.26 | 4.06 | 14.93 | 3.01 | 25.96 | 0.00 | 7.10 | 3.17 | 10.26 |
| Mn | 0.30 | 0.00 | 0.37 | 0.00 | 0.67 | 0.58 | 1.81 | 8.17 | 29.33 | 32.93 | 72.83 | 3.79 | 31.24 | 6.43 | 41.47 |
| Co | 0.22 | 0.00 | 0.54 | 0.00 | 0.75 | 0.96 | 2.16 | 19.77 | 0.00 | 0.00 | 22.89 | 0.00 | 0.00 | 0.00 | 0.00 |
| Ni | 0.00 | 0.00 | 0.00 | 0.00 | 0.00 | 0.85 | 0.00 | 2.76 | 12.15 | 0.00 | 15.76 | 2.91 | 0.00 | 0.00 | 2.91 |
| Cu | 0.00 | 0.00 | 0.00 | 0.00 | 0.00 | 3.06 | 2.40 | 15.58 | 4.87 | 10.79 | 36.70 | 7.16 | 2.77 | 6.86 | 16.80 |
| Zn | 0.39 | 0.00 | 0.00 | 0.79 | 1.18 | 0.00 | 6.31 | 46.99 | 192.99 | 244.19 | 490.48 | 11.32 | 45.20 | 80.61 | 137.13 |
| Ge | 0.00 | 0.00 | 0.00 | 1.24 | 1.24 | 0.00 | 0.00 | 0.00 | 15.99 | 0.00 | 15.99 | 15.17 | 0.00 | 4.85 | 20.02 |
| As | 0.00 | 0.00 | 0.00 | 2.34 | 2.34 | 5.13 | 2.68 | 0.00 | 0.00 | 15.48 | 23.29 | 0.00 | 0.00 | 0.00 | 0.00 |
| Se | 0.00 | 0.00 | 0.00 | 1.65 | 1.65 | 0.00 | 0.00 | 0.00 | 0.00 | 12.75 | 12.75 | 0.00 | 32.98 | 0.00 | 32.98 |
| Br | 0.00 | 0.00 | 0.00 | 0.00 | 0.00 | 0.00 | 0.00 | 0.00 | 10.62 | 0.00 | 10.62 | 3.42 | 32.70 | 0.00 | 36.12 |
| Hg | 0.00 | 0.00 | 0.00 | 0.00 | 0.00 | 0.00 | 0.00 | 0.00 | 0.00 | 86.62 | 86.62 | 0.00 | 112.50 | 0.00 | 112.50 |
| Pb | 0.00 | 0.00 | 0.00 | 7.43 | 7.43 | 0.00 | 0.00 | 0.00 | 0.00 | 0.00 | 0.00 | 38.24 | 0.00 | 0.00 | 38.24 |
